# Factors associated with knowledge of a Good Samaritan Law among young adults who use prescription opioids non-medically

**DOI:** 10.1186/s12954-016-0113-2

**Published:** 2016-07-26

**Authors:** Tristan I. Evans, Scott E. Hadland, Melissa A. Clark, Traci C. Green, Brandon D. L. Marshall

**Affiliations:** 1 Department of Epidemiology, Brown University School of Public Health, 121 South Main Street, Box G-S-121-2, Providence, RI 02912 USA; 2 Division of Adolescent/Young Adult Medicine, Department of Medicine, Boston Children’s Hospital, 333 Longwood Avenue, Boston, MA 02115 USA; 3 Department of Pediatrics, Harvard Medical School, 25 Shattuck Street, Boston, MA 02115 USA; 4 Department of Health Policy and Management, Harvard T. H. Chan School of Public Health, 677 Huntington Ave, Boston, MA 02115 USA; 5 Department of Quantitative Health Sciences and Center for Health Policy and Research, University of Massachusetts Medical School, 55 Lake Ave North, Worcester, MA 01605 USA; 6 Department of Emergency Medicine, Boston University School of Medicine, 771 Albany Street, Room 1208, Boston, MA 02118 USA; 7 Rhode Island Hospital, The Warren Alpert School of Medicine of Brown University, 55 Claverick Street, Providence, RI 02903 USA

**Keywords:** Good Samaritan Law, Opioid, Non-medical prescription opioid, Young adult, Overdose

## Abstract

**Background:**

To date, no studies have examined the extent of knowledge and perceptions of Good Samaritan Laws (GSLs) among young adults who engage in non-medical prescription opioid (NMPO) use. We sought to determine awareness of and factors associated with knowledge of Rhode Island’s Good Samaritan Law (RIGSL) among young adult NMPO users.

**Findings:**

We compared the sociodemographic and overdose-related characteristics of participants who were aware and unaware of the RIGSL and determined independent correlates of knowledge of the RIGSL via modified stepwise logistic regression. Among 198 eligible participants, 15.7 % were black, 62.1 % white, and 20.7 % mixed or other race. The mean age was 24.5 (SD = 3.2) and 129 (65.2 %) were male. Fewer than half (45.5 %) were aware of the RIGSL; nonetheless, the majority (95.5 %) reported a willingness to call 911 in the event of an overdose. Knowledge of the RIGSL was associated with older age, white race, a history of incarceration, a history of injection drug use, lifetime heroin use, ever witnessing or experiencing an overdose, having heard of naloxone, knowledge of where to obtain naloxone, and experience administering naloxone (all *p* < 0.05). In the final explanatory regression model, lifetime injection drug use, having heard of naloxone, and knowledge of where to obtain naloxone were independently associated with awareness of the RIGSL.

**Conclusions:**

Fewer than half of NMPO users surveyed knew of the RIGSL. Targeted harm reduction education is needed to address a vulnerable population of NMPO users who have not initiated injection drug use and are unaware of naloxone. Additional research is needed to determine how the effectiveness of GSLs could be improved to prevent overdose deaths among young adults.

## Introduction

Since the early 2000s, non-medical prescription opioid (NMPO) use and heroin use have resulted in dramatically escalating rates of unintentional drug overdose across the USA [1, 2]. In response to the ongoing nationwide opioid epidemic, a number of US states have implemented harm reduction programs and legislative policies focused on reducing the occurrence of fatal opioid overdose, including prescription monitoring programs, laws restricting new prescriptions for opioid medications, training programs for opioid users to recognize overdose, and equipping first responders and community outreach organizations with naloxone [3–5]. As of 2015, 34 US states and the District of Columbia had also enacted limited opioid amnesty laws [6]. The objective of such laws is to encourage persons who witness an overdose to contact emergency personnel, even if there are controlled substances at the scene. Rhode Island’s Good Samaritan Overdose Prevention Act, R.I. Gen. Laws §21-28.8-4 (2012), first enacted in 2012 and reinstated in 2016, grants limited legal immunity to anyone who seeks or receives medical assistance for a drug-related overdose or medical emergency, unless that person is involved with the manufacture and/or distribution of controlled substances. Specifically, the law protects “Any person who, in good faith, without malice and in the absence of evidence of an intent to defraud seeks medical assistance for someone experiencing a drug overdose or other drug-related medical emergency shall not be charged or prosecuted for any crime under RIGL 21–28 or 21–28.5, except for a crime involving the manufacture or possession with the intent to manufacture a controlled substance or possession with intent to deliver a controlled substance, if the evidence for the charge was gained as a result of the seeking of medical assistance” [7]. Notably, the 2016 version of the law provides immunity to individuals on probation or parole (i.e., seeking medical assistance in the event of an overdose is not a violation of the terms of probation/parole).

Young adults who engage in NMPO use are a high-risk subpopulation of opioid users [8, 9]. They are often in the process of developing long-lasting and potentially life-altering patterns of polysubstance and opioid use; furthermore, young adults who engage in casual experimentation with NMPO use may transition to heroin and injection drug use [10–12]. Many harm reduction services, including syringe exchange and overdose education programs, have primarily targeted adult and/or NMPO users who use heroin and/or inject drugs [3, 13–18]. Therefore, young opioid users who are experimenting with NMPO use are at high risk for overdose but may have received less harm reduction education and, as a result, may not know how to react to an overdose and may be unaware of the legal protections afforded by Good Samaritan Laws (GSLs) [19].

Frequently skeptical of authority (in light of the often punitive legal consequences associated with heroin and opioid possession), young adults also face perceived and systemic deterrents that may prevent them from contacting emergency personnel in the event of an opioid overdose [20]. To date, no studies have examined the extent of knowledge and perceptions of GSLs among young adult NMPO users. This study represents an initial assessment of the awareness of Rhode Island’s GSL (RIGSL), R.I. Gen. Laws §21-28.8-4 (2012), among 18–29-year-old NMPO users. Due to the socially and legally sensitive nature of the raw data, the minimum eligible age (18 years) was selected in order to include the perspectives of the youngest possible adults able to autonomously provide legal consent (in the state of Rhode Island) to participate in a sensitive public health research study. To be consistent with previously published research [21, 22], we defined the maximum age limit for the term “young adult” to be 29 years of age. The primary objective of the analysis was to assess the sociodemographic and behavioral factors associated with knowledge of the RIGSL.

## Methods

Participants were recruited from January 2015 through February 2016 through online venues (e.g., Craigslist), bus advertisements, word of mouth, flyers, and referrals. Eligible participants were Rhode Island (RI) residents between the ages of 18 and 29 who had engaged in NMPO use within 30 days prior to their interview. NMPO use was defined as consuming a prescription opioid that was not prescribed by a licensed medical provider, or as using a prescription opioid with a different mode of administration (e.g., snorted, injected) or dosage than was prescribed. Surveys included questions about drug-related risk behavior, sexual history, employment and housing status, and NMPO and polysubstance use. A trained interviewer administered all survey questions except those related to injection drug use, which were administered via computer-assisted personal interviewing (CAPI). Participants received $25 upon completing the hour-long survey. Between January 2015 and June 2015, we attempted to recruit participants through respondent-driven sampling (RDS). The RDS approach was terminated in June 2015 due to the limited referral of eligible participants from their peers who completed the survey; no other peer drug users were involved in the recruitment of study participants.

This study received approval from a research ethics committee, the Brown University Institutional Review Board (IRB #1403001006), and conforms to the standards outlined in the Declaration of Helsinki. Participants voluntarily signed informed consent forms prior to completing a confidential survey.

The primary outcome relevant to this analysis was: “Have you ever heard of Rhode Island’s Good Samaritan Law?” Descriptive statistics and frequencies were used to compare the characteristics of the participants who were aware and unaware of the RIGSL. Pearson’s *χ*^2^ tests and Fisher’s exact tests (for any cell counts <5) were used to determine associations between knowledge of the RIGSL and the variables listed in Table 1, which are related to injection drug use, heroin and NMPO use, experienced and witnessed opioid overdose, and naloxone access and harm reduction knowledge. We also assessed the willingness of participants to recruit emergency services by asking “Would you call 911 if you or someone you knew was overdosing, even if drugs were present at the scene?” prior to asking them if they were aware of the RIGSL. Finally, among participants who had overdosed in the last 6 months, we examined the actual actions that bystanders had taken to mitigate instances of opioid overdose (e.g., calling 911, administering naloxone).

**Table 1:** Factors associated with knowledge of the RIGSL among young adult NMPO users (*n* = 198)

Knowledge of Rhode Island’s GSL	Total *n* (%)	Aware *n* (%)	Unaware *n* (%)	*p* value
Mean age (years, SD)	198 (100)	25.3 (2.96)	23.9 (3.32)	0.003^a^
Sex				
Male	129 (65.2)	64 (49.6)	65 (50.4)	0.145
Female	69 (34.8)	26 (37.7)	43 (62.3)	
Ethnicity				
Not Hispanic or Latino	170 (85.9)	77 (45.3)	93 (54.7)	0.911
Hispanic or Latino	28 (14.1)	13 (46.4)	15 (53.6)	
Race				
Black, African, Haitian, or Cape Verdean	31 (15.7)	9 (29.0)	22 (71.0)	0.012
White	123 (62.1)	66 (53.7)	57 (46.3)	
Aggregated “all other races”	41 (20.7)	14 (34.1)	27 (65.9)	
Education				
Some high school or less	23 (11.6)	8 (34.8)	15 (65.2)	0.558^b^
Finished high school or GED	75 (37.9)	34 (45.3)	41 (54.7)	
Some college	74 (37.4)	35 (47.3)	39 (52.7)	
Trade or technical school	7 (3.5)	5 (71.4)	2 (28.6)	
College or university degree	19 (9.6)	8 (42.1)	11 (57.9)	
History of incarceration				
Yes	73 (36.9)	52 (55.9)	41 (44.1)	0.008
No	105 (53.0)	38 (36.2)	67 (63.8)	
History of homelessness				
Yes	108 (54.5)	57 (52.8)	51 (47.2)	0.034
No	90 (45.5)	33 (36.7)	57 (63.3)	
Lifetime injection drug use				
Yes	59 (29.8)	43 (72.9)	16 (27.1)	<0.001
No	138 (69.7)	46 (33.3)	92 (66.7)	
Lifetime heroin use				
Yes	85 (42.9)	55 (64.7)	30 (35.3)	<0.001
No	113 (57.1)	35 (31.0)	78 (69.0)	
Ever witnessed an overdose				
Yes	102 (51.5)	55 (53.9)	47 (46.1)	0.020
No	96 (48.5)	35 (36.5)	61 (63.5)	
Ever experienced an overdose				
Yes	53 (26.8)	32 (60.4)	21 (39.6)	0.017
No	145 (73.2)	58 (40.0)	87 (60.0)	
Have you ever heard of Narcan™, otherwise known as naloxone?				
Yes	120 (60.6)	75 (62.5)	45 (37.5)	<0.001
No	78 (39.4)	15 (19.2)	63 (80.8)	
Do you know where to buy or obtain Narcan™?				
Yes	82 (41.4)	60 (73.2)	22 (26.8)	<0.001
No	116 (58.6)	30 (25.9)	86 (74.1)	
Have you ever administered Narcan™ to someone you thought was overdosing?				
Yes	21 (10.6)	16 (76.2)	5 (23.8)	0.006
No	177 (89.4)	74 (41.8)	103 (58.2)	
Would you call 911 in the event of an overdose, even if there were drugs at the scene?				
Yes	189 (95.5)	87 (46.0)	102 (54.0)	0.729^b^
No	8 (4.0)	3 (37.5)	5 (62.5)	

^a^Welch’s *t* test

^b^Fisher’s exact test

We used a modified backward stepwise regression procedure [23, 24] to build an explanatory multivariate model and to identify variables independently correlated with knowledge of the RIGSL. In brief, variables listed in Table 1 with *p* < 0.20 were included in the initial model (variables in Table 2 were not considered due to low response counts). The covariate with the highest type III *p* value was eliminated in subsequent rounds of regression. The model with the lowest Akaike information criterion (AIC) was selected as the final model. R version 3.2.3(C) 2015 was used for all statistical analysis, and all *p* values were two-sided.

**Table 2:** Responses to recent overdoses and knowledge of the RIGSL among young adult NMPO users who reported experiencing an overdose in the past 6 months (*n* = 21)

Knowledge of Rhode Island’s GSL	Total *n* (%)^a^	Aware *n* (%)	Unaware *n* (%)
The last time you overdosed, were you alone?			
Yes	4 (19.0)	1 (25.0)	3 (75.0)
No	17 (81.0)	13 (76.5)	4 (23.5)
Did someone call 911 the last time you overdosed?^b^			
Yes	6 (28.6)	6 (100.0)	0 (0.0)
No	11 (52.4)	7 (63.6)	4 (36.4)
The last time you overdosed, were you taken to a hospital?			
Yes	8 (38.1)	7 (87.5)	1 (12.5)
No	13 (61.9)	7 (53.8)	6 (46.2)
The last time you overdosed, did someone administer naloxone?			
Yes	12 (57.1)	9 (75.0)	3 (25.0)
No	6 (28.6)	4 (66.7)	2 (33.3)
Who administered the naloxone (the last time you overdosed)?^c^			
A police officer	4 (19.0)	3 (75.0)	1 (25.0)
An ambulance official	2 (9.5)	2 (100.0)	0 (0.0)
A friend	3 (14.3)	2 (66.7)	1 (33.3)
A parent or relative	1 (4.8)	0 (0.0)	1 (100.0)
Someone else	2 (9.5)	2 (100.0)	0 (0.0)

Note: “Recent overdoses” were those that occurred within the last 6 months

^a^Not all columns add to 100 % due to missing values

^b^Of those who had experienced an overdose in the past 6 months and were not alone when they overdosed

^c^Of those who had experienced an overdose in the past 6 months and were treated with naloxone

## Results

Out of 200 total recruited participants, 198 (99 %) answered the question “have you ever heard of Rhode Island’s Good Samaritan Law?” and were included in the analysis. The mean age of the sample was 24.5 years (SD = 3.2), 129 (65.2 %) were male, and 14.1 % identified their ethnicity as Hispanic or Latino. In addition, the eligible sample was 15.7 % black (African American or of African, Haitian, or Cape Verdean descent), 62.1 % white, and 20.7 % were of mixed or other race.

Among eligible participants, 90 (45.5 %) reported awareness of the RIGSL. Knowledge of the RIGSL varied by race: 53.7 % of white, 29.0 % of black, and 34.1 % of other/mixed race participants reported being aware of the RIGSL (*p* = 0.012). Other sociodemographic factors associated with awareness of the RIGSL included older age, prior incarceration, and history of homelessness (see Table 1). A sub-analysis of drug use behaviors by race showed that white participants had the highest prevalence of lifetime heroin use (61.8 versus 11.1 % for black and mixed/other race participants, *p* < 0.001), lifetime injection drug use (44.3 versus 5.6 % for black and mixed/other race participants, *p* < 0.001), and history of overdose (36.6 versus 9.7 % for black and mixed/other race participants, *p* < 0.001).

A trend emerged in which participants with more extensive drug use profiles were (proportionally) more aware of the RIGSL. For example, of the 59 NMPO users who had ever injected drugs, 72.9 % were aware of the RIGSL, whereas only 33.3 % of non-injectors were aware of the RIGSL (*p* < 0.001). This trend held for heroin use, with 64.7 % of participants who had tried heroin and only 31.0 % who had not tried heroin reporting awareness of the RIGSL (*p* < 0.001). Having ever experienced an overdose and having ever seen someone overdose were also both associated with awareness of the RIGSL (both *p* < 0.05, see Table 1).

Several measures of harm reduction education (e.g., having ever heard of naloxone, knowing where naloxone could be obtained, and having ever administered naloxone) were significantly and positively associated with knowledge of the RIGSL (all *p* < 0.05). In addition, the vast majority of participants (95.5 %) reported a willingness to call 911 in the event of an overdose-related emergency even if there were drugs at the scene. However, among the 189 NMPO users surveyed who reported willingness to call 911, only 46.0 % were aware of the GSL, and knowledge of the GSL was not associated with a willingness to call 911 (*p* = 0.729).

The final explanatory multivariate model with the lowest AIC included the following covariates: history of injection drug use (APR = 1.29, 95 % CI 1.01–1.57, *p* = 0.064), having heard of naloxone (APR = 1.88, 95 % CI 1.28–2.49, *p* = 0.071), and knowledge of where to obtain naloxone (APR = 1.73, 95 % CI 1.29–2.17, *p* = 0.002).

Lastly, among the 21 participants who experienced an opioid overdose within 6 months of completing the survey, 14 (66.7 %) were aware of the RIGSL. Of note, all six individuals who reported having an individual call 911 the last time they overdosed were aware of the RISGSL. However, we have no data indicating that the individuals who actually dialed 911 in response to these (six) particular overdose situations were aware of the RIGSL. Of the 12 individuals who reported naloxone being administered the last time they overdosed, 9 (75.0 %) were aware of the RIGSL. Other reactions to individuals’ most recent overdose stratified by knowledge of the RIGSL are shown in Table 2.

## Discussion

In this study of young adult NMPO users in RI, knowledge of the RIGSL was moderately high but unevenly distributed across the study population. Despite this, participants reported nearly universal willingness to recruit 911 in the event of an opioid emergency. Knowledge of the RIGSL was less common among young NMPO users who had not used heroin, injected drugs, or witnessed or experienced an overdose.

Given these findings, it is possible that young adult NMPO users in RI first learn about the RIGSL after experiencing or witnessing an overdose. A second possible explanation is that harm reduction programs (which provide naloxone education, overdose training, and injection equipment) may educate their high-risk clientele about the protections afforded by GSLs. In fact, knowledge of where naloxone could be obtained was the strongest correlate of awareness of the RIGSL. Other covariates included in the final model (history of injection drug use, having heard of naloxone) also support the assertion that young NMPO users in RI (and likely also their opioid using peers) may currently be receiving education about the RIGSL through harm reduction outlets that provide naloxone and injection equipment.

Over 95 % of the study participants reported willingness to call 911 in the event of an overdose, despite the fact that fear of arrest has historically been a primary reason for failing to contact emergency medical services during an overdose [25]. In RI, a recent study showed high levels of knowledge of RIGSL among police officers and near universal awareness of the protections afforded by the RIGSL among those who received opioid overdose training [26]. It is possible that these educational and outreach efforts with law enforcement personnel, and as well considerable media coverage encouraging individuals to contact emergency services in the event of an overdose, have led to a high level of willingness to call 911 in this setting.

In light of these findings, it is important to consider how the RIGSL could be made more effective, even when NMPO-using young adults are aware of the GSL. For example, the physiological consequences of opioid overdose may render individuals who overdose alone physically unable to recruit help or emergency services. It follows that the effectiveness of the RIGSL likely depends, in part, on the degree to which individuals engage in social (or monitored) NMPO use. Of the 21 participants who reported a recent overdose, 4 (19.0 %) were alone. Thus, interventions may be needed to encourage young adults who use prescription opioids non-medically to avoid doing so alone, in addition to campaigns promoting the protections afforded by GSLs.

A recent report showed that targeting naloxone education programs to nearly 3000 bystanders (individuals who might have contact with NMPO and heroin users) successfully decreased the rate of fatal opioid overdose in 19 towns across Massachusetts [3]. Given these results, it may be prudent to target education about the RIGSL to bystanders (i.e., close friends and family of young NMPO users), as well as to young NMPO users themselves, in order to improve the efficacy of the RIGSL.

This analysis should be considered in light of several limitations. First, underreporting of socially undesirable behaviors (e.g., ever using heroin, ever injecting, being unwilling to call 911 in response to a potentially fatal overdose) is possible. Second, the small sample size impeded robust examination of the relationship between knowledge of the RIGSL and the actions participants took in response to recent overdoses. Finally, although multiple online and field-based methods were used to recruit study participants, the findings may not necessarily be generalizable to all young adult NMPO users in RI. Unfortunately, to our knowledge, no other studies have examined NMPO use among young adults in RI or other states in the New England region. Future studies are needed to confirm whether this sample is representative of the target population.

In sum, we observed moderate levels of awareness of a GSL among young adults who use prescription opioids non-medically in RI. Targeted harm reduction education (concerning the RIGSL but also general overdose prevention and response training) may be needed to address a vulnerable population of NMPO users who have not initiated injection drug or heroin use. Additional research is needed to determine how the effectiveness of GSLs could be improved to prevent overdose deaths among young adults.

## Abbreviations

GSL, Good Samaritan Law; NMPO, non-medical prescription opioid; RI, Rhode Island; RIGSL, Rhode Island’s Good Samaritan Law
